# A *de novo* KCNQ2 Gene Mutation Associated With Non-familial Early Onset Seizures: Case Report and Revision of Literature Data

**DOI:** 10.3389/fped.2019.00348

**Published:** 2019-09-06

**Authors:** Gianluigi Laccetta, Simona Fiori, Matteo Giampietri, Annarita Ferrari, Valentina Cetica, Manuela Bernardini, Francesca Chesi, Sara Mazzotti, Elena Parrini, Massimiliano Ciantelli, Andrea Guzzetta, Paolo Ghirri

**Affiliations:** ^1^Division of Neonatology and Neonatal Intensive Care Unit, Department of Maternal and Child Health, Santa Chiara Hospital, University of Pisa, Pisa, Italy; ^2^Department of Developmental Neuroscience, IRCCS Stella Maris, Pisa, Italy; ^3^Pediatric Neurology, Neurogenetics and Neurobiology Unit and Laboratories, Neuroscience Department, Meyer Children's University Hospital, University of Florence, Florence, Italy; ^4^Department of Clinical and Experimental Medicine, University of Pisa, Pisa, Italy

**Keywords:** KCNQ2, seizures, mutation, benign familial neonatal convulsions, phenobarbital

## Abstract

Among neonatal epileptic syndromes, benign familial neonatal seizures (BFNS) are often due to autosomal-dominant mutations of the KCNQ2 gene. Seizures are usually characterized by asymmetric tonic posturing with apnea with onset in the first 7 days of life; they may even occur more than 10 times per day or evolve into status epilepticus. The delivery course of our patient was uneventful and family history was negative; on the second day of life the baby became pale, rigid, and apnoic during breastfeeding and appeared jittery and irritable when stimulated or examined. At age 3 days, she experienced clusters of generalized tonic seizures with pallor, desaturation, bradycardia, and partial response to intravenous phenobarbital; during her 4th and 5th days of life, three episodes of tonic seizures were noticed. At age 6 days, the patient experienced about 10 episodes of tonic seizures involving both sides of the body, which gradually responded to intravenous phenytoin. Electroencephalograms revealed abnormalities but brain MRI was normal. The patient is seizure-free since postnatal day 21; she is now 12 months old with cognitive development within normal limits at Bayley III Scale and mild motor delay. The patient is on maintenance therapy with phenobarbital since she was 7 months old. A *de novo* heterozygous mutation (c.853C>T/p.P285S) in the KCNQ2 gene was identified. We therefore describe a case of *de novo* KCNQ2-related neonatal convulsions with necessity of multiple anticonvulsants for the control of seizures, mutation occurring in the pore channel of the voltage-gated potassium channel subfamily Q member 2 associated with a likely benign course; furthermore, the same mutation of the KCNQ2 gene and a similar one (c.854C>A/p.P285H) have already been described in association with Ohtahara syndrome. Probably acquired environmental, perinatal and genetic risk factors are very important in determining the different phenotype; we hope that the rapid progress of analysis tools in molecular diagnosis can also be used in the search of an individualized therapeutic approach for these patients.

## Introduction

Neonatal seizures are often caused by hypoxic-ischemic encephalopathy, intracranial hemorrhage, hypoglycemia, electrolytes imbalance, and infections; metabolic disorders and neonatal epileptic syndromes occurring in the first week after birth, are rarely involved (1–3).

The KCNQ2 gene encodes for the voltage-gated potassium channel subfamily Q member 2 underlying the M-current; it was firstly described as a cause of autosomal-dominant seizures in 1998 and it is located at 20q13 (4–7). Each KCNQ2 subunit is made up of 6 transmembrane domains (S1–S6) with the voltage-sensor in S4 and the pore region defined by S5, S6, and the intervening linker (4, 8, 9). Mutations of the KCNQ2 gene can result in haploinsufficiency or dominant negative effect (4, 8–11).

Patients carrying KCNQ2 gene mutations will have benign familial neonatal seizures (BFNS), benign familial neonatal-infantile seizures (BFNIS) or benign familial infantile seizures (BFIS); they usually have benign courses even if some affected children will experience recurrent febrile seizures, benign childhood epilepsy with centrotemporal spikes (BCECTS), or photosensitive epilepsy during follow-up (2, 3, 12–15). On the other side, KCNQ2 gene mutations have also been associated with early myoclonic encephalopathy (EME) and early onset epileptic encephalopathy (EOEE), such as Ohtahara syndrome (OS) and intractable partial epilepsy, characterized by poor developmental prognosis (4, 12, 13, 16, 17).

Despite some case series reports and functional studies, the association between the phenotype and the genotype still remains unclear, thus it is not possible to establish prognosis for these patients at the moment of diagnosis (3, 12, 18–20).

Asymmetric tonic posturing and apnea are usually the main features of seizures at onset; they are often followed by unilateral or bilateral clonic jerking (2, 21–23). Seizures may even occur more than 10 times per day or evolve into status epilepticus (2, 21–23). Interictal EEG is usually normal in BFNS but it shows multifocal epileptiform abnormalities, random attenuation, or burst-suppression (BS) pattern in KCNQ2-related encephalopathy: thus, a severely abnormal interictal EEG is important in the differential diagnosis between KCNQ2-related encephalopathy and BFNS (2, 11, 21–23). Patients with KCNQ2-related epilepsies usually present a generalized EEG attenuation or suppression even after brief seizures (2, 17, 22, 23). Brain magnetic resonance imaging (MRI) is usually normal or shows a transient hyperintense globus pallidus (11, 23).

Thus, the suspicion of a KCNQ2-related disorder is mainly based on familial anamnesis, clinical presentation, and EEG pattern; the diagnosis is finally confirmed by direct sequencing of the KCNQ2 gene or targeted next generation sequencing (NGS) panel of epilepsy genes (3, 8).

As regards therapy of KCNQ2-related epilepsies, a recent review states that both phenobarbital and sodium channel blockers (carbamazepine and phenytoin) lead to seizure disappearance in patients with a benign course and patients with EOEE usually achieve seizure freedom when receiving sodium channel blockers (6, 14, 24, 25).

We report a 12-months-old female with a *de novo* KCNQ2 gene mutation presumably associated with non-familial benign neonatal seizures (nfBNS) and investigate the correlation between the genotype and the phenotype revisioning the data of literature. Written informed consent for the publication of this case report was obtained from both the parents of the patient.

## Case Report

This third child of non-consanguineous parents was born at full term by spontaneous vaginal delivery without asphyxia to a 43-year-old woman. The pregnancy course was uneventful; Apgar score was 9 at 5 min and umbilical cord blood gas analysis was normal. Her birth weight was 3,040 g, her body length was 50 cm, and her head circumference was 35 cm. After birth, her condition was good, and she started breastfeeding regularly. Family history was negative for epilepsies, mental retardation and hereditary diseases.

On the second day of life, the infant was noted to become pale, rigid and apnoeic for some seconds while breastfeeding; furthermore she appeared jittery and irritable when stimulated or examined.

At age 3 days, clusters of generalized tonic seizures associated with pallor, desaturation, and bradycardia, were first noticed. The infant was subsequently admitted to the Neonatal Intensive Care Unit (NICU). At first examination, the infant appeared in good general conditions, with normal cardiorespiratory and abdominal examinations. Arterial blood gas and head ultrasound performed upon admission to the NICU were unremarkable. Bedside Amplitude-Integrated EEG (aEEG) monitoring was promptly started and showed a discontinuous normal voltage; during seizures the aEEG trace registered an immediate rise and a subsequent depression of its amplitude. The patient was therefore loaded with intravenous phenobarbital (20 mg/kg), and a maintenance dose of 2.5 mg/kg was administered every 12 h with reduced frequency of seizures but persistent subclinical seizure patterns at aEEG registration. During her 4 and 5th days of life the infant experienced three episodes of tonic seizures with hyperextension of the trunk, right, or left head deviation, involvement of both lower and upper limbs, oral automatisms, and flushing.

In the meantime, laboratory investigations (complete blood count, electrolytes, glucose, plasma lactate and ammonia, plasma amino acids, acyl-carnitines and urinary organic acids, blood culture, viral serology and genomes, markers of infection) and diagnostic procedures (Holter ECG, echocardiogram and abdomen ultrasound scan) yielded no significant results.

At age 6 days the patient had about 10 episodes of tonic seizures which involved both lower and upper limbs and both sides of the body alternately; seizures were accompanied by apnea and turning of head and eyes to one side. Each episode lasted about 30–40 s and persisted despite the administration of intravenous phenobarbital, midazolam (0.2 mg/kg) and pyridoxine (100 mg). Intravenous phenytoin (8 mg/kg/day) was therefore administered from the same day with gradual disappearance of clinically evident seizures on the subsequent days and improvement of the EEG pattern.

First video electroencephalogram (EEG) at 7 days of age showed discontinuous pattern during sleep and spike waves over bilateral frontal and centrotemporal areas with secondary diffusion, predominantly during sleep. The second and third video EEGs (9 and 16 days of age) indicated an improvement of the sleep pattern and the persistence of the spike waves over bilateral frontal and centrotemporal areas during sleep with secondary diffusion.

Brain MRI at 10 days of age was normal as regards ventricular morphology, cortical folding, and white matter intensity; MR spectroscopy was also normal.

Midazolam and pyridoxine were ineffective, thus they were discontinued at 11 days of age. Because of the baby's good general conditions, treatment with oral phenobarbital (2.5 mg/kg every 12 h) and phenytoin (2 mg/kg every 8 h) was started from postnatal day 15 with no further seizures until postnatal day 21 when the infant presented two episodes of generalized tonic seizures involving her limbs with hyperextension of the trunk, left head, and eye deviation and pallor; both episodes were preceded by a high-pitched cry and each lasted about 30–40 s.

Seizures definitively stopped after the administration of intravenous phenobarbital and phenytoin; the patient was seizure free even after the second attempt of switching to oral phenobarbital (2.5 mg/kg every 12 h) and phenytoin (2 mg/kg every 8 h), which was done at 25 days of age.

The fourth video EEG (23 days of age) showed a predominant discontinuous pattern over the right hemisphere during sleep and the persistence of the spike waves over bilateral frontal and centrotemporal areas during sleep with secondary diffusion. The fifth video EEG (30 days of age) indicated a predominant normal background even during sleep with spike waves over bilateral frontal and centrotemporal areas with secondary diffusion more evident on the right side and during sleep.

The following video EEG (37 days of age) showed centro-temporal isolated spikes predominantly on the right hemisphere, with a normal background activity.

At the age of 38 days the patient was discharged from hospital; her general conditions were satisfactory and she was seizure free with oral phenobarbital (5 mg/kg/day in two doses) and phenytoin (6 mg/kg/day in three doses). Neurologic examination before discharge revealed mild generalized hypotonia and poor writhing movements. Based on these findings, a genetically-determined epilepsy of neonatal onset was strongly suspected, therefore genetic testing, including epilepsy NGS panel for 109 genes involved in EOEE (Table 1), was ordered on postnatal day 8.

**Table 1:** Epilepsy NGS panel for 109 genes involved in early epileptic encephalopathies.

ADGRV1	CHRNA2	EPM2A	HCN1	KCTD7	NPRL3	PNPO	SCN8A	SLC35A3
ALDH7A1	CHRNA4	FOXG1	IQSEC2	LGI1	NRXN1	POLG	SIK1	SLC6A1
ALG13	CHRNB2	FOXP1	KCNA1	LIAS	PC	PRICKLE1	SLC12A5	SLC6A8
ARHGEF9	CLCN2	FOXP2	KCNA2	MBD5	PCDH19	PRICKLE2	SLC13A5	SLC9A6
ARX	CNTNAP2	GABRA1	KCNB1	MECP2	PDHA1	PRRT2	SLC19A3	SPTAN1
ATP1A2	CSNK1G1	GABRB3	KCNC1	MEF2C	PDHB	PURA	SLC25A1	ST3GAL3
ATP1A3	CSTB	GABRD	KCNJ10	MPC1	PDP1	QARS	SLC25A12	STX1B
CACNA1A	DEPDC5	GABRG2	KCNK18	MTHFR	PIGA	RELN	SLC25A15	STXBP1
CACNA1H	DLAT	GNAO1	KCNMA1	MTOR	PIGN	SCARB2	SLC25A20	SYNGAP1
CACNB4	DNM1	GRIN1	KCNQ2	NECAP1	PIGT	SCN1A	SLC25A22	SZT2
CASR	DOCK7	GRIN2A	KCNQ3	NHLRC1	PLCB1	SCN1B	SLC2A1	TBC1D24
CDKL5	EFHC1	GRIN2B	KCNT1	NPRL2	PNKP	SCN2A	SLC35A2	UBE3A
CHD2								

After discharge the patient has been followed closely by the Infant Neurology Section at Stella Maris Scientific Institute. At 12 months of age she is seizure-free with progressively reducing doses of phenytoin; from the age of 7 months she is on monotherapy with phenobarbital. Her follow-up neurological examinations reveal a slightly delayed trunk control, sitting position acquired at 8 months of age and mild hypotonia; at 12 months of age she can stand with support, but no walking is possible yet. Video EEG performed at 12 months of age shows spike waves in the frontal-central, centro-parietal and centro-parieto-temporal regions with left predominance. The cognitive subscale of Bayley Scale of Infant Development III performed at 12 months of age shows a normal cognitive development. Communication abilities consist of varied babbling and first words; pointing is present.

DNA samples from the patient and both her parents were extracted from peripheral whole blood by means of a genomic DNA purification kit (Gentra Systems, Minneapolis, MN) after written informed consents were obtained. For the proband, all 17 exons of the KCNQ2 gene were amplified by polymerase chain reaction and sequenced using NexSeq Illumina platform from Illumina (San Diego, CA, USA); the variant detection software used were BWA v0.7.7-r441, Picard v1.109, and GATK v3.1.

A *de novo* heterozygous mutation (c.853C>T, p.P285S) in the KCNQ2 gene was identified in the patient. No mosaicism for the normal variant of the KCNQ2 gene was found in the proband; similarly, no mosaicism for the KCNQ2 variant was identified in the parents. Thus, the family was referred to a genetic counselor: the estimated risk of recurrence was <1% for the parents and 50% for the proband.

## Discussion

In this patient, a heterozygous mutation (c.853C>T, p.P285S) in the KCNQ2 gene was identified, with the consequence of a proline substitution to serine.

The voltage-gated potassium channel Kv7.2, encoded by the KCNQ2 gene, assembles with Kv7.3, encoded by the KCNQ3 gene, to form heteromultimeric channels which regulate neuronal excitability through the M-current (3, 5, 9, 20, 26).

KCNQ2-related epilepsy spectrum ranges from KCNQ2-related BFNS to KCNQ2-related EOEE; KCNQ2 gene mutations have also been linked with other phenotypes such as myokymia associated with neonatal or early infantile epilepsy, peripheral nerve excitability without known epilepsy, fever sensitive epilepsy forms of the generalized epilepsy with febrile seizure plus syndrome and Dravet syndrome (1, 3, 6, 11, 27–31).

Age of onset of seizures strongly correlates with underlying genetics, as most of the non-lesional patients with BFNS/BFNIS show KCNQ2 mutations and those with BFIS commonly show PRRT2 mutations (32).

The baby experienced some episodes of tonic seizures, each one involving one side of the body; these seizure attacks were also accompanied by involvement of both lower and upper limbs, apnea, and turning of head and eyes to one side: these features have already been described in a child with epilepsy of infancy with migrating focal seizures (EIMFS) which evolved to infantile spasms (28). Interestingly, this infant presented a mutation, p.881C>T (p.A294V), in the pore region of the KCNQ2 gene (28).

Furthermore, tonic seizures are more common than generalized tonic-clonic seizures among newborns because of immature myelination (1, 2, 33).

BFNS are usually hereditary, autosomal-dominant seizures occurring in the neonatal age: our patient likely represents a rare case of nfBNS (see Table 2 for further cases).

**Table 2:** KCNQ2 gene mutations associated with non-familial benign neonatal seizures (nf-BNS).

**Mutation**	**Note(s)**	**References**
c.319C>T/p.L107F	-	(34)
c.333_334delGT/p.Ser113HisfsX6	-	(21)
c.683C>T/p.R213W	Associated with c.1241A>G/p.E414G in KCNQ3 gene (inherited from mother; polymorphism in Japanese population)	(35)
c.901G>A/p.G301S	-	(23)
c.910delTTC or TTT/p.F304del	Evolution to BECTS	(36)
c.1065C>G/p.D355Q	-	(23)
c.1657C>T/p.R553W	-	(34)
c.1657C>T/p.R553W	-	(23)
c.1700T>A/p.V567D	-	(21)

In the present case, benign neonatal seizures (BNS) were mainly a clinical diagnosis: analysis of the KCNQ2 gene pointed out a *de novo* heterozygous mutation in our patient (c.853C>T, p.P285S), furthermore it was not possible to accurately predict outcome because the association between mutations of the KCNQ2 gene and subsequent phenotype is unclear and the follow-up of our patient is still short (3).

We firstly hypothesized our patient would have suffered from an epileptic encephalopathy (EE) because of her negative family history for epilepsies, mental retardation and hereditary diseases and because of her seizure semiology at 6 days of age, which was similar to that of EIMFS evolving into epileptic spasms (28). Furthermore, the same mutation of the KCNQ2 gene has already been described in a 8-month-old child with OS (17). This patient experienced tonic and simple partial seizures since his first day of life which persisted despite the administration of phenobarbital, topiramate and valproic acid; his EEG showed a burst-suppression pattern and brain MRI was normal (17). No other data are available about the phenotype of the child. In addition, a similar mutation of the KCNQ2 gene (c.854C>A, p.P285H) has already been described as associated with OS (12). In this case a proline is changed to histidine; the patient experienced irritability with hypoxia since 2 days of age and tonic seizures from 8 days of age (12). Seizures persisted despite the administration of phenobarbital and pyridoxal 5′ phosphate but were controlled by valproic acid; electroencephalograms showed burst-suppression and MRI showed T1- and T2-high signal on globus pallidus at postnatal day 12 and T2-high signal on globus pallidus at 3 months of age (12). The patient was seizure-free since 3 months of age but developed moderate mental retardation and hypotonic quadriplegia; the mutation was maternally inherited and the mother of the patient only suffered from idiopathic epilepsy since neonatal age and she was on treatment with valproic acid (12). Probably acquired environmental, perinatal, and genetic risk factors are very important in determining the different phenotype; as proof of this hypothesis, in one report, a patient with a mutation of c.G1620A (p.K526N) and family history of BNS, developed resistant epilepsy and mental retardation (1, 2, 37). Furthermore, Grinton et al. reported some families with KCNQ2 mutations and family members with the same genotype but different phenotypes (1, 3, 15). Thus, the same gene mutation contributes to different phenotypes; this possibility is valid for many genetic diseases and also for KCNQ2-related diseases (2, 6).

The basal ganglia are susceptible to different stressors, and even more the neonatal brain (3, 6, 11, 38, 39). Basal ganglia are also vulnerable to excitotoxicity caused by frequent neonatal seizures; this might explain the lesions seen in patients with KCNQ2-related epilepsies (3, 6, 11, 40). These lesions are highly specific since they have never been described in association with other severe neonatal epileptic syndromes (OS, EME, or pyridoxine-dependent epilepsy) (6, 11, 24, 27). Our patient had a normal brain MRI at 10 days of age but this result did not exclude EE since many patients with negative brain MRI and EE have already been described (34).

Al Yazidi et al. noted an association between severe neonatal EE phenotype and *de novo* missense mutations of the KCNQ2 gene; even patients with BFNS and missense mutations in KCNQ2, had less benign clinical courses than it is usually predicted, for example they required multiple anticonvulsants for the control of their seizures (3, 11–13, 26). Furthermore, though precise correlation between KCNQ2 gene mutation and phenotype is difficult, BFNS phenotype is usually associated with inherited heterozygous loss-of-function mutations, and EE is usually associated with *de novo* missense mutations (1, 2, 26).

While BFNS-associated variants are distributed throughout the channel protein, currently known KCNQ2 encephalopathy mutations are located in four functionally important protein domains: the voltage sensor domain, the pore, the C-terminus proximal region, and the calmodulin-binding B helix region (2, 7, 8, 11, 41, 42).

Our patient presented the missense mutation c.853C>T (p.P285S) of the KCNQ2 gene, in which a proline is substituted by a serine; this change, presumably leading to a reduction of current potassium or other abnormal functional properties/subcellular localization of Kv7.2 channel, is responsible for the phenotype of our patient in association with additional genetic, perinatal and environmental factors (2, 9, 10).

The mutation has been classified as pathogenic according to the American College of Medical Genetics (ACMG) Scoring System, deleterious at SIFT analysis and damaging at Mutation Taster analysis, thus confirming previous findings (17).

The mutation might be responsible for the phenotype of the child even because no other pathogenic or likely pathogenic mutations have been found in the other 108 genes included in the NGS panel for epilepsy. The phenotype of our patient (presumably BNS and not OS) is not explained by an underlying mosaicism for the normal variant of the KCNQ2 gene. The lack of a functional experiment is a limit of the present case report; Miceli et al. drew a genotype-phenotype correlation based on distinct functional properties of Kv7.2 channels carrying different variants on the same residue found in two patients affected by widely divergent epileptic syndromes (43).

Both c.854C>A and c.853C>T are mutations occurring in the portion of the KCNQ2 gene encoding for the pore channel of the voltage-gated potassium channel subfamily Q member 2; other mutations involving the pore region have already been described (Table 3).

**Table 3:** Review of the KCNQ2 gene mutations involving the pore region of the voltage-gated potassium channel subfamily Q member 2.

**Nucleotide/protein changes**	**Phenotype**	**Inheritance**	**Seizures onset**	**Semiology of seizures**	**EEG appearance at onset**	**First brain MRI**	**Medications trialed**	**Drug control**	**Outcome**	**References**
c.761_770del10insA in KCNQ2 gene + c.2687A>G (p.N821S) in KCNQ3 gene	BFNC (patient, mother, sister), West syndrome (maternal aunt)	c.761_770del10insA in KCNQ2 gene: maternally inherited; c.2687A>G in KCNQ3 gene: paternally inherited	3rd day	C seizures, right head and eyes rotation, palpebral myoclonias, oro-alimentary automatisms, and cyanosis	Fast polyspikes on the left centro-temporal areas with diffusion; normal organization background	Normal	PB	PB	SF. At 18 months of age normal neurological examination and psychomotor development	(44)
c.766G>T p.G256W	EE									(45)
c.773A>G p.N258S	BFNC									(46)
c.775G>T p.D259Y	BFNC	Inherited	3rd day	Eye rolling, joint stiffness, and upper limb clonus	Normal	Normal	PB, VA	PB, VA	SF. Normal psychomotor development	(34)
c.790T>A p.Y264N	Seizures during neonatal period and infancy									(47)
c.793G>A p.A265T	EE	*De novo*	Before 2nd day	T and/or C, T with apnea, ES, F hypermotor	Bursts of asynchronous spikes and sharp waves, discontinuity/BS	T1 hypersignal of the pallida, caudate nuclei, and hyppocampi; T2 hypersignal of the parietal occipital white matter/Normal	PB, MDZ, LVT, PH, ZNS, VA, VGB, CLB, BTN, FLT, KD, TPM, B6, ACTH, EZO	Improvement with EZO	M jerks /ES/SF since early adolescence. Poor psychomotor development	(27), (18), (42), (34)
c.793G>C p.A265P	EE	*De novo*	7th day	T flexion spasms	Multifocal epileptic activity	Small frontal and temporal lobes, thin CC, hyper-T2, and hypo-T1 signals in cerebellum, frontal ventriculomegaly, increased frontotemporal extra-axial CSF spaces	VGB, VA, TPM	VGB temporary response	SF since age 2 y 6 m. Poor psychomotor development	(11)
c.794C>T p.A265V	EE	*De novo*	1st-5th day	Apneic spells, T spasms with right opsoclonus-like movement, T seizures, left-sided seizures	BS/ Multifocal sharp waves	Mildly delayed myelination, T2 high signal on GP/Normal	CBZ, DZP, MDZ, PB, PLP, VA, B6, ZNS, CLN, CLB, VGB	DZP, MDZ, PB partially effective; CBZ effective	Myoclonus at the bilateral upper extremities/SF/Intractable seizures. Poor psychomotor development	(13), (12)
c.802C>T p.L268F	EE	*De novo*	1st day	F seizures	Multifocal epileptiform discharges	Diffuse hypomielination with volumetric reduction of the frontal lobe	PB, PH, LVT, TPM	LEV+TPM	Mild global impairment	(24)
c.803T>C p.L268P	EE (Ohtahara-type), multifocal epilepsy	*De novo*	<24 hrs of life	T, TC, and F seizures	BS	Delayed myelination	PB, LVT, PH, TPM, OXC, BTN, FLT	OXC+TPM	SF. Poor psychomotor delay	(34)
p.W269L	EE						EZO	Improvement with EZO		(42)
c.807G>A p.W269Ter	BFNC	Inherited	3rd day	F, initial T phase followed by asynchronous C phase	Normal background with centrotemporal sharp waves		CBZ	CBZ	SF. Normal psychomotor development	(21)
c.807G>A p.W269X	BFNC, febrile seizures, generalized epilepsy in adulthood									(9)
c.811C>T p.A294V	EE		1st day		Discontinuity, asymmetric low voltage suppression, interictal epileptiform discharges, poorly organized background	Small focus of reduced diffusion in the left posterior parietal white matter	PB, LVT, B6, TPM, PH	PB, LVT, TPM	Reemergence of seizure activity upon attempt to wean AED	(48)
c.812G>T p.G271V	BFIC, choreoathetosis, myokymia									(30)
c.821C>T p.T274M	EE	*De novo*	2nd-3rd day	Stiffening, head, and eye deviation, T posturing, T and hypoT seizures, ES	BS, hypsarrhythmia, right temporal asymptomatic seizures	Hyperintensity of basal ganglia, irregular right thalamus, small frontal lobe, reduced white matter volume, thin CC, giant perivascular spaces/Normal	LVT, PB, PH, CLN, VGB, GBP, TPM, PRD, VA, OXC, EZO, CLB	OXC; some improvement with TPM; SF, reduced frequency of ES, increased alertness, and tone with EZO	SF/ES. Poor psychomotor development	(11), (27), (42), (34)
c.827C>T p.T276I	EE (Ohtahara-type)	*De novo*	1st day		BS	Reduced posterior white matter volume, thin CC			Poor psychomotor development	(49)
c.830C>T p.T277I	EE	*De novo*	2nd day	T seizures, FC activity	DNV	Normal	PB, MDZ, PN, LVT, LDN, CLN, TPM, VA	LVT and LDN acutely effective, VA effective	SF since 1.5m, one febrile seizure at 1y. Poor psychomotor development	(23)
c.835G>T p.G279C	EE								Intellectual disability	(50)
c.841G>A p.G281R	EE	*De novo*	2nd day	T, T spasm-like, M, and hemiC seizures	BS	Atrophy of frontal lobe, thin CC, delayed myelination	PB, VGB, VA, PH, TPM, LVT, LTG, KD, ZNS	PB transient response, KD some response	Daily M seizures, clusters of T seizures, poor psychomotor development	(18)
c.841G>C p.G281R	EE	*De novo*	1st day	T, C seizures	BS	Small thalami	PB, KD, RTG, TPM, steroids, VGB, VA, LVT, LCM	PB+KD+ high dose RTG (strong reduction in seizures); TPM and steroids some response	Weekly T seizures. Poor psychomotor development	(18)
c.841G>T p.G281W	EE	*De novo*	2nd day	SE, T asymmetric seizures	Multifocal epileptiform discharges	Hyperintensity in the basal ganglia, thalami/hippocampus	PB, LVT, CBZ	CBZ	Poor psychomotor delay	(24)
c.847_848insGT p.K283SfsTer36	BFNC	Inherited								(5)
c.850T>C p.Y284H	EE	*De novo*	<1 m of life	F, M, G seizures; spasms					SF. Poor psychomotor development	(51)
c.850T>G p.Y284D	EE	*De novo*	48 hrs	F seizures, startle episodes, ES, T seizures	Hypsarrhythmia	Cortical-subcortical atrophy with altered white matter, thin CC	PB, PN, VA, VGB, ZNS, CLB, CBZ, LVT	None	ES, T seizures. Poor psychomotor development	(34)
c.851A>G p.Y284C	BFNC									(5)
c.854C>A p.P285H	OS	Inherited (mother: idiopathic epilepsy since neonate, medicated with VA)	2nd day	T seizures	BS, asymmetric	T1 and T2 high signal on GP	VA, PLP, PB	VA	SF. Poor psychomotor development	(12)
p.P285S	OS	*De novo*	1st day	T, simple partial seizures	BS	Normal	PB, TPM, VA	None	Refractory epilepsy	(17)
c.860C>A p.T287N	EE		1st day	C, T seizures	BS	Normal	PB, VGB		SF. Poor psychomotor development	(27)
c.868G>A p.G290S	EE	*De novo*	1st day	T seizures	BS, multifocal slow waves, frontal, and occipital spikes, generalized flattening	Normal	PB, VGB, CBZ, PH, LDN, CLN	PH	SF. Poor psychomotor development	(12), (27)
c.869G>A p.G290D	EE	*De novo*	2nd day	T; myoclonias of arms and eyelid, C movements	BS/ Multifocal epileptic activity	Thin CC/ Hyperintensity of basal ganglia	PB, LVT, VA, CLN	VA+CLN	Monthly T or TC seizures, often with fever; SF since then. Poor psychomotor development	(11)
c.875T>C p.L292P	EE		1st day		Discontinuity with epochs of complete suppression in either or both hemisphere and epileptiform discharges, poorly organized background	Normal	PB, LVT, TPM, CBZ	CBZ, LVT	At the 9 m follow-up visit the patient was meeting the developmental milestones. SF but difficulty in balancing seizure control with sedation and feeding	(48)
c.881C>G p.A294G	BFNC									(52)
c.881C>T p.A294V	Mostly EE; EIMFS evolving to IS	Mostly de novo	10 h-14th day	Excessive paroxysmal fetal movements; neonatal jitteriness; M, T, C, GTC seizures; spasms; SE; startle episodes	BS; hypsarrhythmia, multifocal/bilateral discharges, attenuation, discontinuity, asynchrony, slow background activity	Normal/ Increased signal over lentiform nuclei bilaterally/ T2 high signal on GP	PB, LVT, MDZ, VA, PH, TPM, VGB, CLN, BTN, FLT, PDN, ACTH, B6, CBZ, ZNS, PLP, NZP	PH (initial response), LVT (minimal response), ACTH (partially effective), VA, CBZ, ZNS, TPM	SF/ Uncontrollable seizure. Poor psychomotor development	(53), (12), (27), (54), (52), (24), (34), (28)

BFNS-causing mutations are often non-sense, whereas OS/EOEE-causing mutations are all missense, as previously described by Millichap et al. (42).

*AED, anti-epileptic drugs; BFIC benign familial infantile convulsions; BFNC, benign familial neonatal convulsions; BS, burst suppression; BTN, biotin; B6, vitamin B6; C, clonic; CBZ, carbamazepine; CC, corpus callosum; CLB, clobazam; CLN, clonazepam; DNV, discontinuous normal voltage; DZP, diazepam; EE, epileptic encephalopathy; EIMFS, epilepsy of infancy with migrating focal seizures; ES, epileptic spasms; EZO, ezogabine; F, focal; FC, focal clonic; FLT, folate; G, generalized; GP, globus pallidus; GBP, gabapentin; hr, hour; IS, infantile spasms; KD, ketogenic diet; LCM, lacosamide; LDN, lidocaine; LTG, lamotrigine; LVT, levetiracetam; M, myoclonic; m month; MDZ, midazolam; NZP, nitrazepam; OS, Ohtahara syndrome; OXC, oxcarbazepine; PB, phenobarbital; PDN, prednisone; PH, phenytoin; PLP, pyridoxal 5' phosphate; PN, pyridoxine; RTG, retiagabine; SE, status epilepticus; SF, seizure-free; T, tonic; TC, tonic-clonic; TPM, topiramate; VA, valproic acid; VGB, vigabatrin; wks, weeks; y, year; ZNS, zonisamide*.

Even though neonatal convulsions constitute a “benign” epileptic syndrome, our patient showed clusters of seizures with apnea, desaturation and bradycardia, which required second-line drugs to achieve seizures control. After the administration of intravenous phenobarbital, seizures showed reduced frequency; at age 6 days the patient experienced about ten episodes of tonic seizures which persisted despite the administration of intravenous phenobarbital, midazolam and pyridoxine, and gradually disappeared after the administration of intravenous phenytoin. At age 21 days, while on treatment with oral phenobarbital and phenytoin, the infant experienced two episodes of generalized tonic seizures which stopped after the administration of intravenous phenobarbital and phenytoin. At 25 days of age, the patient was therefore switched to oral phenobarbital and phenytoin with no further recurrence of seizures; she is now seizure-free on oral treatment with phenobarbital.

The pattern of BNS involves apnea, cyanosis, automatisms, focal clonic seizures and, rarely, generalized tonic-clonic seizures; some patients experience recurrence of seizures after 1 year of age (most commonly febrile seizures, generalized seizures with febrile seizures-plus, or BCECTS) (1, 2). For all these reasons, the diagnosis of neonatal seizures can be difficult and genetic testing becomes mandatory; furthermore, seizures semiology made clinical diagnosis more difficult for our patient. We would have preferred to start therapy with oral carbamazepine immediately because it is safe and highly effective for BNS and it permits to avoid sedation, hypotonia and delayed oral feeding associated with intravenous phenobarbital and phenytoin but it requires prompt recognition of the electroclinical phenotype; furthermore, our patient presented clusters of seizures with apnea, desaturation, and bradycardia requiring a rapidly effective intravenous therapy (21).

Our patient will stop oral therapy with phenobarbital within a few months because the risk of recurrence of seizures is minimum when medications are weaned between 12 and 18 months of life (7, 21, 55). Unfortunately, up to 25% of neonates with BNS may experience other forms of epilepsy later in childhood and mental retardation occurs in rare cases, therefore our infant is strictly followed up (2, 7, 8, 15, 21, 37).

## Data Availability

The datasets generated for this study can be found in UO Neonatologia AOUP.

## Ethics Statement

The work involves a human subject and it has been conducted in conformity with ethical standards of the field and with consent of both parents of the child. Written informed consent for the publication of this case report was obtained from both the parents of the patient.

## Author Contributions

Study concept and design: GL, PG, SF, and AG. Patient collection: PG, FC, MB, MG, MC, AF, SF, AG, and SM. Mutation screening: VC and EP. Data analysis and review: GL and PG. Drafting of the manuscript: GL and PG. Critical revision of the manuscript for important intellectual content: PG, SF, and AG. Obtained funding: PG.

### Conflict of Interest Statement

The authors declare that the research was conducted in the absence of any commercial or financial relationships that could be construed as a potential conflict of interest.
